# Eliciting meta consent for future secondary research use of health data using a smartphone application - a proof of concept study in the Danish population

**DOI:** 10.1186/s12910-017-0209-6

**Published:** 2017-08-15

**Authors:** Thomas Ploug, Søren Holm

**Affiliations:** 1 Centre for Applied Ethics and Philosophy of Science, Department of Communication, Aalborg University Copenhagen, A C Meyers Vænge, 2450 Kbh. SV, Aalborg, Denmark; 2 Centre for Social Ethicse and Policy, School of Law, University of Manchester, M13 9PL, Manchester, UK; 3 Center for Medical Ethics, Faculty of Medicine, University of Oslo, Oslo, Norway; 4 Centre for Applied Ethics, Aalborg University, Aalborg, Denmark

**Keywords:** Blanket consent, Broad consent, Meta consent, Proof of concept, Specific consent, Secondary research use of health data, Smartphone application

## Abstract

**Background:**

The increased use of information technology in every day health care creates vast amounts of stored health data that can be used for research. The secondary research use of routinely collected data raises questions about appropriate consent mechanisms for such use. One option is meta consent where individuals state their own consent preferences in relation to future use of their data, e.g. whether they want the data to be accessible to researchers under conditions of specific consent, broad consent, blanket consent or not at all.

This study investigates whether meta consent preferences can be successfully elicited by a smartphone application in the adult Danish population.

**Methods:**

A smartphone app was developed for the elicitation of meta consent preferences. An invitation to use the app was distributed to a stratified, representative sample of the Danish adult population. The meta consent choices, the use of the app, user experience data, and demographic data were logged and analysed statistically using IBM SPSS version 20.

**Results:**

Of 1000 potential respondents 221 used the app. One hundred eighty-eight of the respondents were female and 103 male. The age range was 19 to 79 years with an average of 51 years (SD 16). Most users indicate 1) that they find the choices they are asked to make easy to understand (>75% find it ‘Easy’ or ‘Very easy’), 2) that the application is easy to use (>75% find it ‘Easy’ or ‘Very easy’), and 3) that this kind of choice should be offered to people (89% find it ‘Absolutely’ or ‘Somewhat’ important).

**Conclusions:**

It is possible to collect meta consent preferences in the general, adult population using a smartphone app.

**Electronic supplementary material:**

The online version of this article (doi:10.1186/s12910-017-0209-6) contains supplementary material, which is available to authorized users.

## Background

### The meta consent model

The pervasive use of information and communication technology in the health care sector creates new opportunities for research under the banner of ‘Big Data’ [1, 2]. Vast amounts of data collected and stored by different health care institutions may potentially be linked and analysed, and may also be linked to other databases holding non-health related information. A key ethical concern is whether data collected as part of health care can be used for ‘secondary’ research without renewed consent [3–12].

A requirement of informed consent cannot be waived, it may be argued, for at least four reasons: 1) It allows individuals to express approval or disapproval of the purpose of – including the values, methods, interests inherent in – a research project. 2) It allows individuals to protect themselves against harms ensuing from the use of their data, including e.g. stigmatisation of their peer group and medicalization. 3) It allows individuals to protect their privacy by controlling the use of what they consider to be private and sensitive information. 4) It protects trust in health care professionals by involving people in decisions about the use of their data.

However, if a requirement of informed consent is maintained for every single, specific research project in which individuals’ health care data is used it may impede valuable research by imposing on researchers the practical burden of obtaining consent. And, even worse, it may lead to the routinisation of informed consent, i.e. that consent is being provided or refused as an unreflective, habitual act [13, 14]. This may happen as a result of individuals being asked too often about consent. If the provision or refusal of consent becomes routinised – i.e. provided or refused out of habit and without reflection – it clearly loses its ability to protect individuals and their interests as sketched in 1)-4) above.

One solution to this apparent dilemma may be found in the notion of ‘meta consent’ [15, 16]. Meta consent denotes the idea that individuals should be asked how and when they would like to provide consent. As such meta consent is a matter of designing future consent requests. The design process essentially consists in an individual choosing 1) a type of consent for each 2) type of data or type of context, from a set of predefined options. Table 1 shows a non-exhaustive list of possible categories for 1) and 2):

**Table 1:** Types of consent, data and contexts

Type of consent	Type of data or context
Specific consent: Consent to specific research projetcs	Electronic Patient Record
Broad consent: Consent to broad categories of research	Data from samples
Blanket consent: Consent to all research	Commercial research
Blanket refusal: Refusal of all research use	…

An individual choosing ‘specific consent’ for ‘Electronic patient record’ thereby states that s/he wants to be asked for consent to every future research project in which health data from his or her electronic patient record is being used. A choice of ‘blanket refusal’ for ‘commercial research’ means that the individual has refused all future use of his or her health data in a commercial context. In an implementation of meta consent individuals should be able to change these meta consent choices whenever they want to do so, and should be reminded to revisit their meta consent choices at regular intervals.

A model of meta consent is sensitive to individual preferences for how and when to provide consent, and this sensitivity may increase the likelihood of individuals reflecting upon their future provision or refusal of consent. At the same time a model of meta consent allows for broad and blanket consent, thereby potentially alleviating the practical burden on researchers and reducing the risk of routinisation.

However, while meta consent seem a neat solution in principle, it is far from obvious that ‘meta choices’ of this kind works in practice. In this article we report and analyse the results of a proof of concept implementation of meta consent as a front end application for smartphones and tablets. We investigate 1) whether Danish citizens are able to use a meta consent app, 2) whether they understand the choices presented, 3) how they use the app, and 4) whether they think that the choices are valuable. It is important to note that in Denmark it is currently legal to perform research on health data without consent if the data are anonymised before they are provided to the researchers. Such research does not require research ethics approval either. This situation is likely to continue after the implementation of the EU General Data Protection Regulation (GDPR) in 2018. Article 89 of the GDPR allows for national derogation from consent requirements for use of data for scientific research purposes, and Denmark is likely to use this possibility. An introduction of meta consent will thus in the Danish context allow people more control of their data than they currently have.

The practical Implementation of meta consent also requires a back end. That is a technological infrastructure at the societal level that enables the collection of meta consent, the storage of meta consent choices, the generation of specific consent requests depending on the meta choices, and the two way communication between individuals and researchers of specific consent requests and decisions. This could be implemented relatively easily in countries where individuals are identifiable through a unique personal identification code, and where citizens are already required to have a publicly authorised electronic mailbox (in Denmark and Norway, for example). In these countries, the personal identification code is already used to link data for epidemiological research and to direct mail to the mailbox. Implementing a further link to a consent request generator would be straightforward [15].

### The current study and recent research on informed consent

Two relevant strands in recent discussions on informed consent should be mentioned here.

The model of meta consent presents only one possible strategy in the attempt to overcome the problem of routinisation and consent fatigue. Recent literature on informed consent has stressed the importance of tailoring the ongoing information and communication about research to individual informational needs by empowering individuals to take part in the design of the information exchange through a dynamic, web-based platform [17, 18]. Key in the discussion of these different models of informed consent is the question of whether a choice to provide broad or blanket consent for future research can be informed since it cannot be founded on specific information about specific research projects [19–24]. If a choice to provide broad of blanket consent cannot be informed, this will obviously pose a problem for any consent model allowing broad or blanket consent, including the meta consent model. We believe that such choices can be informed in the sense that they can rest on general information about future research, and this information may well in many cases satisfy individual preferences for information. And, if it does not satisfy individual preferences for information, the model of meta consent provides individuals with the option of requiring to be asked for consent to each specific, future research project on the basis of specific information about these projects. However, in this article we do not aim to give an exhaustive treatment of strengths and weaknesses of alternative models of consent but rather to present the results of an empirical study of an electronic implementation of meta consent.

A second relevant strand in recent literature on informed consent concerns the feasibility of implementing informed consent processes in a digital setup. Several digital models and solutions have been suggested in the scientific literature [25–28], and different commercial electronic solutions have emerged (Secure Consent, WCG eConsent, iCONS eConsent [29–31]). Common to these are that they aim to make the consent process easier for patients in order to increase patient participation in the consent process. The current study also focuses on an electronic implementation of consent. It differs from previous studies and solutions in two respects. First, it does not concern consent for a specific clinical trial, or other specified research project but rather consent for all future secondary use of health data collected in the clinical context. Second, and relatedly, it primarily studies meta preferences for consent, i.e. it studies peoples’ preferences for how and when to provide consent in relation to predefined categories of research.

## Methods

### App development

The application was developed on the basis of four initial criteria: 1) Users should be provided with information about the collection and potential research use of their health care data in the Danish health care sector, and they should be given information about the purpose of the application. 2) Users should be given an explanation of the four types of consent outlined above. 3) Users should be given information about the various types of data and research contexts and then be provided with the possibility to choose the format of future consent requests. The types of data reflect the most common data types held in Danish registries, and the types of research the types most commonly mentioned in Danish public debate. Moving beyond a proof of concept study would require a much more rigorous process for choosing and defining these types, and they might differ from country to country. 4) Users should be provided with an overview of their choices and an estimate of how their consent choices would a) affect them in terms of the number of future consent requests, and b) limit the research use of their data. A choice of specific consent for data types and contexts would thus entail many future consent requests to the individual and a significant limitation of research, whereas a choice of blanket consent to all data types and contexts would entail no future consent requests to the individual and no limitation of research.

**Fig. 1:**
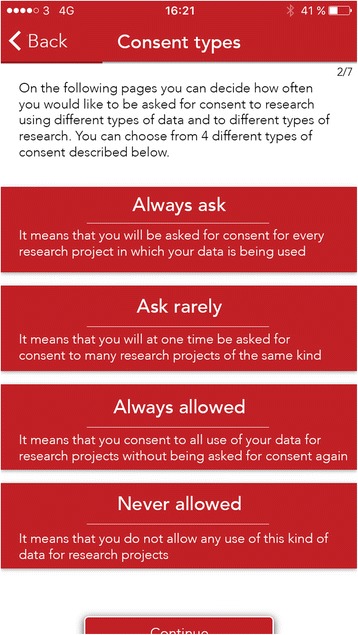
Consent Types. The four different types of consent

**Fig. 2:**
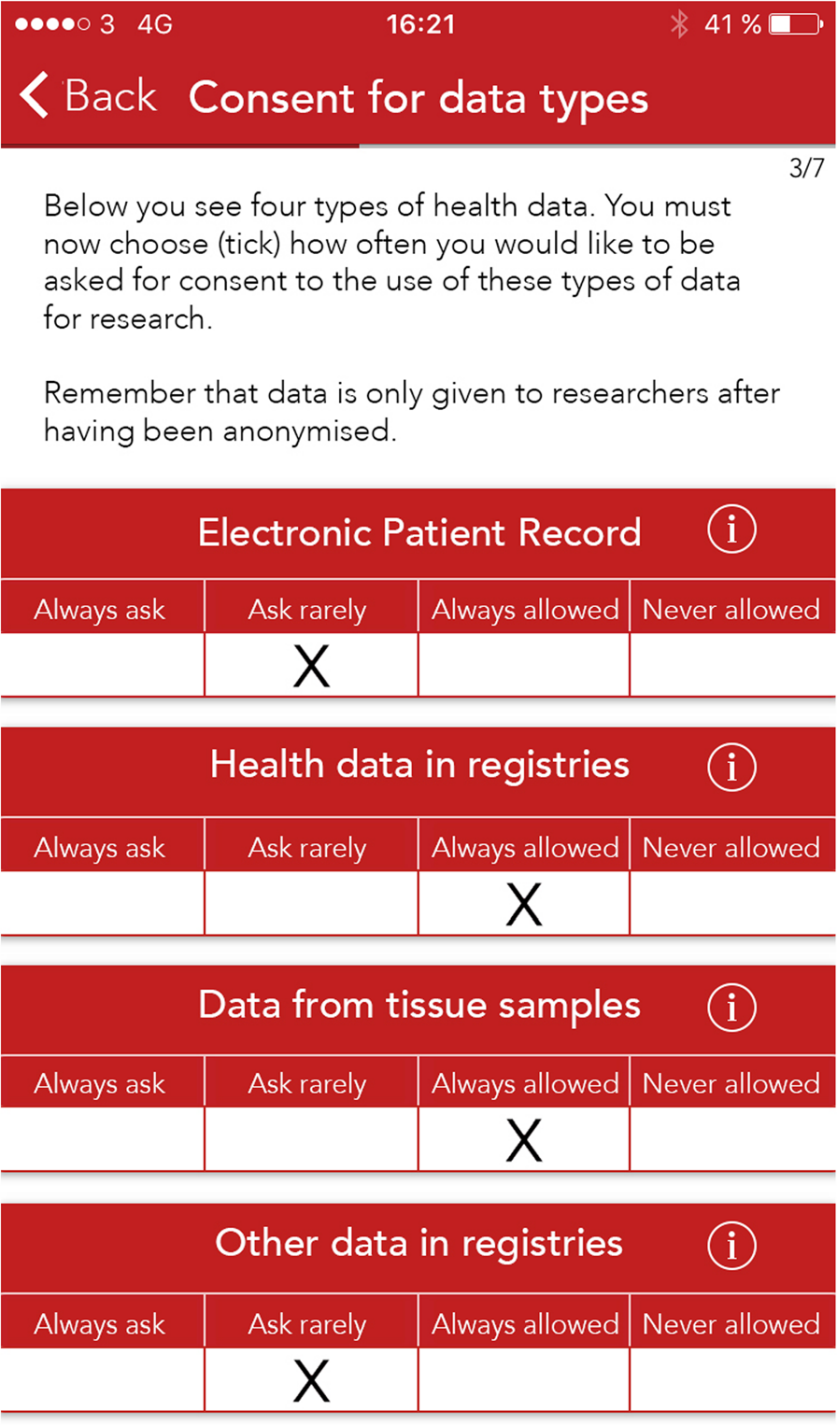
Consent for Data Types. An example of meta consent

These four criteria along with common user interface considerations formed the basis for the user-centred development of a paper prototype, a mockup and the final application for Android and iOS platforms by professional app developing company Syntac Studios. Figures 1 and 2 below show two of the main screens in the final application (Additional file 1 contains a translation from Danish of all the screens in the app including the ‘Help’ screens).

User expectations to content and layout of the paper prototype and the image-based mockup prototype was tested on 6 and 8 subjects. They were asked “To what degree does the contents and layout of the screen fulfill your expectations?” Using a Likert scale from 1 to 5 with 1 being “Horrible” and 5 “Perfect”, the paper prototype and the image-based mockup prototype scored an average per screen of 3.5-4.1 and 4.25-4.5 respectively. The first full application prototype was tested on 31 subjects using the standard extensively validated, 10 item System Usability Scale and scored an average 75 on a scale from 1 to 100 [32]. A score of 68 or above indicates that a system is easy to approach and intuitive [33]. Participants for these tests where recruited through an e-mail to students and employees of the Department of Communication, Aalborg University, Copenhagen, and through Syntac Studio’s list of contacts.

### Sample

A random, stratified sample of 1000 potential respondents, representative of the adult Danish population was drawn from TNS Gallup’s Danish panel. The sample was stratified according to gender, age and residential region. The background panel consists of approximately 53,000 persons. Potential respondents were contacted by e-mail by TNS Gallup, with further reminders by e-mail and SMS to those who had not either 1) stated that they did not have a suitable smartphone or tablet, or 2) declined participation. An incentive for participation was provided in the form of entry into a prize draw for a number of vouchers with a value of 300 DKK.

### Analysis

Data from the app were captured by a server back end and exported to SPSS IBM Statistics version 20 and analysed using the SPSS table and regression functions [34].

### Ethics

The study collected anonymous data only and therefore does not require research ethics approval in Denmark. Participation is completely voluntary and the questions asked are not particularly sensitive or likely to cause distress.

## Results

An invitation to participate in the study was sent by e-mail to 1000 potential respondents in May 2016. One hundred eighty-eight were excluded because they did not have a smartphone or tablet and 2 for other reasons. Four hundred seven did not wish to participate. Of the remaining 403 potential participants, 305 activated the link to the app and 221 used and completed the app. The response rate is thus 22.1% of the total sample, and 27.2% of the sample having smartphones or tablets. Compared with the stratification variables (Gender, Age and Region) the sample efficiency was 89% (Sample Efficiency = ${n}^2\div n{\sum}_i{w}_i^2\Big)$.

One hundred eighteen of the respondents were female and 103 male. The age range was 19 to 79 years with an average of 51 years (SD 16). The educational level of the respondents were ‘7-10 years of school’ 35 (15.8%), ‘11-13 years of school’ 65 (29.4%), ‘Short university education (less than 3 years)’ 27 (12.2%), ‘Medium university education (3–4.5 years)’ 70 (31.7%), ‘Long university education (5 years or more)’ 24 (10.9%). The educational level in the general Danish population 15 to 69 years old in 2016 were ‘7-10 years of school’ 16.3%, ‘11–13 years of school’ 39.7%, ‘Short university education’ 6.4%, ‘Medium university education’ 13.6%, ‘Long university education’ 7.4%, and ‘Not known’ 5.6% (Data extracted from Statistics Denmark, Statistikbanken, Disced-15 classification of highest educational level) [35].

The respondents’ assessment of the difficulties of understanding the choices they had to make is presented in Table 2, and their actual choices in Table 3.

**Table 2:** Perceived difficulty of choices

	1 Very difficult	2	3	4	5 Very easy
How easy was it to understand the different types of consent?	0^a^	9 (4.1)	29 (13.1)	58 (26.2)	125 (56.6)
How easy was it to understand the different types of data and types of research?	2 (0.9)	14 (6.3)	41 (18.6)	70 (31.7)	94 (42.5)
How easy was it to understand the choices you were asked to make?	1 (0.5)	16 (7.2)	33 (14.9)	60 (27.1)	111 (50.2)
How easy was it to use the app?	1 (0.5)	0	10 (4.5)	40 (18.1)	170 (76.9)

*N* = 221 ^a^ *n* (%)

**Table 3:** Meta consent preferences

Type of data	Blanket refusal (Never allowed)	Specific consent (Ask always)	Broad consent (Ask rarely)	Blanket consent (Always allowed)
Electronic patient record	1 (0.5)^a^	90 (40.7)	39 (17.6)	91 (41.2)
Health data in registries	2 (0.9)	74 (33.5)	43 (19.5)	102 (46.2)
Data from samples	1 (0.5)	83 (37.6)	40 (18.1)	97 (43.9)
Other data in registries	2 (0.9)	97 (43.9)	47 (21.3)	75 (33.9)
Type of research				
Public research	3 (1.4)	66 (29.9)	52 (23.5)	100 (45.2)
Private commercial research	9 (4.1)	137 (62.0)	41 (18.6)	34 (15.4))
Private non-commercial research	4 (1.8)	113 (51.1)	52 (23.5)	52 (23.5)
Danish research	4 (1.8)	79 (35.7)	54 (24.4)	84 (38.0)
International research	7 (3.2)	121 (54.8)	46 (20.8)	47 (21.3)

*N* = 221 ^a^ *n* (%)

The movements of the respondents through the app were logged and the time used on each of the main screens is shown in Table 4. The distributions of times are skewed to the right and we therefore present median and quartiles. The respondents could click on information bubbles to get more information about data types and research types. Thirty-two percent of respondents sought further information at least once.

**Table 4:** Time use on app pages

Times in seconds	Consent types information page	Data types consent choice page	Research types consent choice page	Your choices confirmation page	Total time in app^a^
Minimum	1.2	1.2	1.4	1.2	48.4
25% percentile	22.7	25.3	19.5	7.7	195.6
Median	32.0	39.2	30.0	17.8	263.5
75% percentile	47.8	65.4	47.7	31.6	380.9
Maximum	263.0	236.3	281.2	506.2	1293.8

*N* = 221 ^a^The times for the individual pages do not sum to the total time, since the app contains an introductory and a thank you page, a demographic questionnaire, as well as optional further information pages

We used multivariate linear regression analysis to explore the associations between the time used in the app as the dependent variable and age, gender and an ad hoc “Ease of use” scale constructed by adding the responses to the four questions about perceived difficulty of choices (Table 2) as the independent variables. The analysis shows a statistically significant, but weak positive association between age and time used in the app (Model R [2] = 0.061, p(age) = 0.006, Beta(age) = 0.227), and no significant associations with either gender or the Ease of use scale in the multivariate model.

After having made and confirmed their choices respondents were asked how important it is that citizens in Denmark are provided with the opportunity to make these choices with answers on a 5 point Likert scale from “Absolutely not important” to “Absolutely important”. 149 (67.4%) found it to be absolutely important, 48 (21.7%) somewhat important, 16 (7.2%) expressed a neutral opinion, and 8 (3.6%) found it somewhat unimportant. No respondent stated that providing these choices to citizens was absolutely not important.

## Discussion

The response rate is low. The good sample efficiency indicates, however, that the respondents do not differ markedly from the initial sample with respect to the three stratification variables: gender, age, and residential region. The respondents have a slightly higher educational level than the average Danish population, and it is likely that the people who used the app are more able and willing to use IT technology than the average Danish citizen. The results may therefore to some extent overestimate how well the choices are understood and how easy the app is to use. The results never the less indicate that the respondents feel that they understand the choices they are asked to make and that the app in itself is easy to use. The mean age of respondents of 51 years indicates that the app was not only tested by ‘young digital natives’.

Subjectively reported understanding and ease of use are important in order to maintain the users’ motivation to engage in consent procedures. Thus the results show that it is possible to design an informed consent app that does not undermine motivation. Objectively establishing understanding is important in order to secure that the relevant consent procedures actually provide the individual with control and protect individual interests. Future testing of meta consent should therefore not only include self-reported understanding but also objective measures of understanding.

The consent choices made strongly indicate that there are significant differences in consent preferences within the Danish population. Very few do not want their data used for any kind of research, and many are willing to let their data be used without specific consent, especially in the case of public research. However the majority consistently across data and research types want some control over the use of their data. This latter point is also substantiated by the answers to the question concerning whether Danish citizens should be provided with the opportunity to make meta consent choices. Underneath these general trends are found noteworthy differences in the consent preferences. Around twice as many want to be asked for specific consent for commercial research and for international research than for public research. The number of people wanting to be asked for specific consent impacts research. A high number will increase the practical burden of obtaining consent for every research project, and it may also indicate a level of scepticism towards the relevant type of research that may eventually lead to more people refusing consent for specific projects of the relevant type. The latter effect may lead to consent bias [36, 37]. These effects on research should, however, be seen in light of the general trends that very few refuse consent to all research use of data, and a high number of people are willing to provide broad or blanket consent to research. And, the potential effects on research must be weighed against the previously listed reasons for protecting individuals through a requirement of informed consent (avoiding harm, protection of autonomy, privacy and trust). The wider discussion of how research and societal interests should be balanced against individuals’ interests is outside the scope of this article.

People who had a prior interest in the issues surrounding biomedical research may have been more likely to participate in the testing of the app, which may also skew the actual choices made. The very low level of respondents choosing blanket refusal to all use of their data, however seems to indicate that people with very strong views against research are not overrepresented in the sample.

The results concerning the time used in the app is consistent with the overall finding that the users find it easy to understand the information provided and to use the application. There is no reason to believe that the ‘median user’ did not take the task seriously, and the median time of approximately 4.5 min indicate that information about consent types, types of data and types of research may be processed and choices made within a very reasonable amount of time. However, the results clearly also indicate that some respondents probably did not take the task sufficiently seriously. In this study it was made clear to respondents that this was only a test of the application, and that their choices had no real life effects. In a real implementation of a meta consent system citizens would be aware that they made real choices of significant importance and would therefore be likely to take the task more seriously.

If a meta consent model is officially introduced it will very likely be accompanied by a major public information campaign about the available choices. This might change peoples’ perception of some or all of the choices, so the results from this study can only provide an indication of what meta consent choices the Danish population might make. It is, however highly unlikely that further information about the research use of health and other data would change the view of all of the respondents and move them into the category of those persons who are willing always to let their data be used without any kind of prior consent. Meta consent choices are revisable, and it is also likely that some people will modify their initial choices in light of the consent requests they will receive or not receive. Some may want to restrict the frequency consent requests and others may want to receive requests for particular types of data or research more often.

## Conclusion

This study has shown that it is possible to elicit meta consent preferences, i.e. preferences for future consent to secondary use of data for research, through the use of a specifically designed smartphone application. Most users indicate 1) that they find the choices they are asked to make easy to understand, 2) that the application is easy to use, and 3) that this kind of choice should be offered to people.

The study also shows that people have significantly different consent preferences, thus underscoring the need for a nuanced consent system such as meta consent.

The study thus provides proof of concept for the use of a front end meta consent application used by citizens.

## Additional file

Additional file 1: The appendix contains a translation of each of the screens in the meta consent application. (DOCX 19 kb)
