# Supplementary material for: Eliciting meta consent for future secondary research use of health data using a smartphone application - a proof of concept study in the Danish population
Source: BMC Med Ethics. 2017 Aug 15;18:51. doi: 10.1186/s12910-017-0209-6 (PMC5558710; doi:10.1186/s12910-017-0209-6)
Supplement: Additional file 1: — The appendix contains a translation of each of the screens in the meta consent application. (DOCX 19 kb) [file 12910_2017_209_MOESM1_ESM.docx]

Additional file 1 Translation of Text in Meta Consent App

**FRONTSCREEN**

Consent for Health Research.  

Tell us how you would like to give consent to the use of your data in health research in the future.

**SCREEN 1:** Consent in health research
Data about your health is collected routinely every time you are in contact with the health care services and is used for many purposes including administration and planning.

These data can also be used for research either alone or together with data from other registries. In this app you can choose how often you would like to be asked for consent to the use of your health data for research in the future.

You can read more about data and research below. If you want to move straight on from here, all you have to do is to press the button “continue”.

What is health data?
Health data is data about your health. They are collected at the doctor, in a hospital or in other places where you are in contact with the health care services. It may also be tissue, e.g residual blood samples or tissue removed during surgery.

What is health care research?
Health care research is research aimed at improving treatments or prevention of diseases. For example by giving us a better understanding of how diseases develop and how treatments work. A type of health care research relies specifically on the use of health data and tissue. The use of health data and tissue for research is regulated by the Research Ethics Commitees and the Data Protection Agency.

How will my choices affect me?
Your choices are important for two interrelated things: 1) How many times you in the future will be asked for content to the use of your data and your tissue, and therefore 2) the extent to which you control the use of your data and your tissue. The choices, that you and others make, will also affect how easy it will be to do health research in Denmark.

**SCREEN 2:** Consent types
On the following pages you can decide how often you would like to be asked for consent to research using different types of data and to different types of research. You can choose from 4 different types of consent described below.

Always ask
It means that you will be asked for consent for every research project in which your data is being used.

Ask rarely
It means that you will at one time be asked for consent to many research projects of the same kind.

Always allowed
It means that you consent to all use of your data for research projects without being asked for consent again

Never allowed

It means that you do not allow any use of this kind of data for research projects.

**SCREEN 3:** Consent for data types

Below you see four types of health data. You must now choose (tick) how often you would like to be asked for consent to the use of these types of data for research.

Remember that data is only given to researchers after having been anonymised.

Always ask -------- Ask rarely -------- Always allowed -------- Never allowed

Electronic Patient Record (i)

(i) Contains data about you medical history, treatment, contact with health care services etc. You have an electronic patient record at your GP, dentist or if you have been in contact with a hospital. The record contains all the information that you have provided or that has been collected as part of medical examinations etc.

Health data in registries (i)

(i) Information about you is routinely collected whenever you are in contact with the health care services. This information is stored in central registries (databases) such as the National Patient Registry which contains information about all treatments in hospitals.

Data from tissue samples (i)

(i) Tissue samples are collected and stored in connection with bloodsampling and surgery. Your tissue contain information that may tell something about possible genetic diseases and about conditions that gives you a risk of certain diseases.

Other data in registries (i)

(i) Information about a wide variety of your personal circumstances – for instance about education, income, jobs, housing, etc. – is routinely collected by public authorities. In combination with health data this information can be used for health research, e.g. research on links between environment and disease.    

**SCREEN 4:** Consent types of research

Below you see five types of health research. You must now choose (tick) how often you would like to be asked for consent to the use of your data for these types of research.

Remember that data is only given to researchers after having been anonymised.

Always ask -------- Ask rarely -------- Always allowed -------- Never allowed

Public research (i)

(i) Public research is research conducted by public institutions such as universities or hospitals.

Private commercial research (i)

(i) Private commercial research is research done by private companies such as pharmaceutical companies.

Private non-commercial research (i)
(i) Private non-commercial research is research done by private organisations such as patient organisations.

Danish research (i)
(i) Danish research is research conducted by Danish institutions.

International research (i)
(i) International research is research conducted by foreign institutions.

**SCREEN 5:** Your Choices
Your choices are listed below. They imply that is likely that you will receive (a lot/some/few/none) requests for consent. Your choice also means that you in certain areas have restricted the use of your health data (a lot/some/little/completely).

If you are satisfied with your choices, click the button below. If you want to change your choices, you can go back and change them.

Types of data Choice

Electronic Patient Record (Always ask/Ask rarely/Always allowed/Never Allowed)

Health data in registries

Data from tissue

Other data

Types of research

Public research

Private commercial research

Private non-commercial research

Danish research

International research
